# Quasiparticle dynamics by effective $\pi$-field distortion

**DOI:** 10.1038/s41598-022-11832-2

**Published:** 2022-05-13

**Authors:** Tiago de Sousa Araújo Cassiano, Pedro Henrique de Oliveira Neto, Geraldo Magela e Silva

**Affiliations:** Institute of Pysics, University of Braília, Brasília, Brazil

**Keywords:** Electronic properties and materials, Two-dimensional materials

## Abstract

Modeling dynamical processes of quasiparticles in low dimensional $\pi$-conjugated systems is challenging due to electron-phonon coupling. We show that this interaction leads to linear potential energy terms in the lattice Lagrangian similar to a local “gravitational” field. The presence of quasiparticles deforms this field in a way analogous to a low-dimension solution of general relativity. Our calculations with analytical expressions for effective $\pi$-fields yield the correct band structure and deliver proper time evolution of the quasiparticle’s properties. Furthermore, we report a sharp reduction in the dynamics computational time up to two orders of magnitude, a result that has major simulation implications.

## Introduction

$\pi$-conjugated systems have been used as primary materials in optoelectronics, being prominent candidates to substitute inorganic devices. Showing several promising properties such as low cost, flexibility, and versatility of synthesis, these materials are already present in photodetectors^35^, organic solar cells^15^, light-emitting diodes^1,12^, and field-effect transistors^16^, among others^34^. Understanding the fundamental properties of organic $\pi$-conjugated compounds constitutes a complex and central problem that can lead to significant technological applications.

Most of the optical and electronic properties of those systems originate from the conjugated bonding based on $\hbox {sp}^2$-hybridized carbons. The combination of strong electron-phonon coupling, intrinsic to $\pi$-electrons, and low dimension confinement effects leads to transport mechanisms mediated by quasiparticles. The dynamics of these carriers determine the spectroscopic, optical, energy, and charge transport properties in $\pi$-conjugated compounds^2^. While excitons are responsible for energy transport, polarons are the main charge carriers. Polarons are lattice distortions that polarize the nearby environment. They are collective excitations with spin 1/2, charge $\pm e$, and effective mass that depends on the material^4,5^. The lattice distortion pattern and the intragap states of the electronic band characterize them. Besides the polaron, other charge carriers may be present in $\pi$-conjugated systems such as bipolarons and solitons, although been less prevalent.

The theoretical description of charge dynamics of $\pi$-conjugated systems is convoluted. Since electron-phonon coupling is present, the electronic and nuclei equations can not be separated solved. They must be solved simultaneously, in a scenario where the Born Oppenheimer approximation does not hold.

Su, Schrieffer, and Heeger were pioneers in modeling the quasiparticle transport by a semi-classical approach for conjugated systems^14,28,30,31^. Their model, known as the SSH model, was initially proposed to describe the conduction in polyacetylene. In the context of a tight-binding method, the core of this model is a first-order approximation of the hopping integral considering lattice distortion. This model was used to describe the morphological and electronic aspects of conjugated polymers carrying quasiparticles. Eventually, further refinements enabled the study of dynamic states^11,13,24,29,33^. The electronic states evolve according to the time-dependent Schrödinger equation, while the lattice does it through Lagrangian mechanics. However, the electron-phonon interaction couples the equations, requiring them to be solved simultaneously. Most of the simulation’s computational cost comes from this constraint.

Recently, the SSH model was extended to 2D systems, increasing even further its applicability and impact. Nowadays, this development is the basis of theoretical research in graphene nanoribbons^3,8,26,27^. In addition, the extension for aggregates, known as the Holstein-Peierls model, became a central tool to investigate intermolecular charge dynamics^9,18,21,23^.

By examining the expected value of the Lagrangian we show that, to the space-time distortion done by mass in general relativity, the polaron is associated with a low-dimensional distortion of the effective $\pi$-force-field. The field of the $\pi$-electrons works as an effective “gravitational” field with the potential energy proportional to the displacement of the carbon atoms. We propose an alternative model to simulate the dynamics of conjugated systems by including this effective potential analytically to describe the $\pi$-electrons influence on the lattice. This change removes the need for higher-order explicit time integration methods. The model’s physical accuracy is examined. We simulated polaron dynamics on cis-polyacetylene with both the conventional model as a reference and our own. Detailed analysis of the polaron drifting process, electronic band structure, energy dynamics, and other phenomena reveals the equivalence between the methodologies. Moreover, the method is also notably cost-efficient, producing time reductions of two orders of magnitude, indicating its use for larger systems in which the conventional procedure becomes impracticable.

## Methods

We present the SSH model in detail to point out precisely what can be done in a different way. Cis-polyacetylene is considered to keep the notation as concise as possible. Besides, its structure is a common backbone of conducting polymers, and the results are extended easily to 2-D $\pi$-systems as graphene nanoribbons. In this formalism, the model Hamiltonian is the sum of electronic ($H_{tb}$) and lattice ($H_{latt}$) terms. The first contribution is a tight-binding-like operator given by^30^

1 $$\begin{aligned} H_{tb} = -\sum _{n,s}[t_{n+1,n}C^{\dagger }_{n+1,s}C_{n,s} + h.c.]. \end{aligned}$$

$C^{\dagger }_{n+1,s}$ is the creation operator of a $\pi$-electron at site $n+1$ with spin *s*. $t_{n+1,n}$ is the hopping integral associated with sites $n+1$ and *n*. The SSH model restricts the physical description to only low-energy excitations^14^. As a result, the $\sigma$-bond fluctuates close to its undisturbed length. The hopping integral is assumed to be dependent on the inter-site distance. Expanding $t_{n+1,n}$ over this length gives

2 $$\begin{aligned} t_{n+1,n} = t_n + \alpha (u_{n+1}-u_{n}), \end{aligned}$$

where $t_n \equiv (1+(-1)^n\delta _0)t_{0}$, with $t_{0}$ the hopping integral in the absence of distortions, $\delta _{0}$ a Brazovskii-Kirova symmetry breaking term, and $u_{n}$ the displacement of site *n* with respect to its equilibrium position. The dependence of the hopping integral with the position is mediated by $\alpha$, the electron-phonon coupling constant.

In addition, the small fluctuation of the *σ*-bonds allows to adopt the harmonic approximation and treat the lattice as a sum of coupled harmonic oscillators,

3 $$ H_{{latt}}  = \frac{K}{2}\sum\limits_{n} {\left( {u_{{n + 1}}  - u_{n} } \right)} ^{2}  + \frac{1}{{2M}}\sum\limits_{n} {p_{n}^{2} } . $$

Here, *K* stands for the elastic constant, *M* is the site’s mass, and $P_{i}$ is the *i*-th conjugated momentum.

It is not possible to diagonalize the electronic Hamiltonian directly since it depends on the lattice configuration. Instead, one must simultaneously solve for both the electronic and lattice in a self-consistent manner^10^.

One begins by finding stationary solutions for the lattice and $\pi$-electrons in an iterative self-consistent way until the convergence criterion is matched. Once the solution converges, it is possible to evolve it in time by including an external electric field. It can be done through a Peierls substitution on the Hamiltonian^25^. Ultimately, this operation only incorporates a time-dependent phase to the hopping integral in Eq. 2, which now reads

4 $$\begin{aligned} t_{n+1,n} = e^{-i\gamma A(t)}(t_n + \alpha (u_{n+1}-u_{n})). \end{aligned}$$

$\gamma \equiv ea/(\hbar c)$, where *e* stands for the elementary charge, *a* the lattice parameter, *c* the speed of light, and *A*(*t*) is the vector potential. The electric field depends only on time, $E(t) = -(1/c){\dot{A}}(t)$.

Let |*ψ*_*k*,*s*_(*t*)〉 be the electronic solution at a given time *t*, and ${\mathbf{U}}(dt) \equiv e^{-iH(t)dt/\hbar }$ the time-evolution operator. Then, the state, after a time interval *dt*, becomes

5 $$\begin{aligned} | {\psi _{k,s}(t+dt)} \rangle = e^{-iH(t)dt/\hbar }| {\psi _{k,s}(t)} \rangle . \end{aligned}$$

Expanding it over the basis of eigenstates, $\{| {\phi _{ls}(t)} \rangle \}$, of the electronic Hamiltonian at a given time *t* leads to,

6 $$\begin{aligned} \begin{aligned} | {\psi _{k,s}(t+dt)} \rangle = \sum _{l}e^{-iE_{l,s}dt/\hbar }| {\phi _{l,s}(t)} \rangle \langle {\phi _{l,s}(t)|\psi _{k,s}(t)} \rangle , \end{aligned} \end{aligned}$$

or,

7 $$ \begin{gathered}   \psi _{{k,s}} (n,t + dt) = \langle n|\psi _{{k,s}} (t + dt)\rangle  \hfill \\ \quad\quad\quad\quad\quad\quad\,\, = \sum\limits_{{l,m}} {\phi _{{l,s}}^{*} } (m,t)\psi _{{k,s}} (m,t)e^{{ - iE_{{l,s}} dt/\hbar }} \phi _{{l,s}} (n,t). \hfill \\  \end{gathered}   $$

As for the lattice, the expected value of the Lagrangian, $\langle {L} \rangle$, obeys the Euler-Lagrange equations,

8 $$\begin{aligned} \frac{d}{dt}\left( \frac{\partial \langle {L} \rangle }{\partial {\dot{u}}_{n}}\right) = \frac{\partial \langle {L} \rangle }{\partial u_{n}}. \end{aligned}$$

$\langle {L} \rangle$ is calculated with a Slater-determinant state composed with all occupied electronic states, $\psi _{k,s}(n,t)$. This equation leads to

9 $$ \begin{gathered}   M\ddot{y}_{n}  = K(y_{{n + 1}}  - 2y_{n}  + y_{{n - 1}} ) \hfill \\   \quad\quad\quad\,\,\, + \alpha (B_{{n + 1}}  - 2B_{n}  + B_{{n - 1}}  + {\mathrm{c}}.{\mathrm{c}}.), \hfill \\  \end{gathered}  $$

with,

10 $$\begin{aligned}  B_{n} \equiv e^{-i\gamma A(t)}\sum _{k,s}\,^{\prime } \psi _{k,s}^{*}(n+1,t)\psi _{k,s}(n,t), \end{aligned} $$

and, $y_{n} = u_{n+1}-u_{n}$. The prime sign means a summation over only occupied states.

The time evolution for the model is determined by equations 7 and 9. When time advances one step, the electronic and lattice configurations change. Because of that, the set $\{\phi _{ls}(t')\}$ is not the same as before. After each time step, the modeling requires the re-diagonalization of the electronic Hamiltonian. Alternatively, one could use high-order explicit time integration methods to evolve the Schrödinger equation. However, these approaches have a significant impact on the algorithm’s computational cost, meaning limitations on system size and simulation time.

Equation 9 is the crucible of $\pi$-systems description. The $B_n$ terms depend on one-electron wavefunctions that are time demanding to find. But, $B_n$ can be calculated analytically for the stationary dimerized configuration^31^. In this case, the results render Eq. 9 as equivalent to a series of connected spring-masses subjected to an alternating gravitational field, since $B_{n+1}=-B_n$. Which can also be deduced by symmetry arguments. Obviously, this picture changes as time and quasiparticles get into consideration. Nevertheless, the change is well-behaved and predictable. It might be more convenient to think in terms of a $\pi$-electrons force field defined by,

11 $$\begin{aligned} \Pi _n = -\alpha (B_n + B_n^*). \end{aligned}$$

Solving numerically in tandem Eqs. 7 and 9 for several systems^5,6,10^, it can be verified that far from quasiparticles and defects, $\Pi _n$ still behaves as an alternating effective “gravitational” field. It is also found that this effective field distorts in association with polarons according to the pattern $sech^2(x-vt)$. It should be pointed out that the square hyperbolic secant dependence could be foreseen from the analytical solutions of the TLM continuum model^32^.

In what follows, we present the simulations of polaron dynamics with the usual approach and the proposed effective field to highlight the equivalence and difference between the two methods.

We set the parameters according to the usual values assigned to conjugated polymers. $\alpha$ = 4.1 eV/Å, $t_{0}$ = 2.5 eV, $\delta _0$ = 0.05, *K* = 21 eV/Å$^{2}$ and *a* = 1.41 Å^19,20^. We simulate a polaron state in the presence of an electric field with a strength of 2.4$\times 10^5$ V/cm. Here, *E*(*t*) will continuously grow from zero until *t* = 50 fs, when it reaches its maximum. Right after, the field drops to 0, remaining off until the end of the simulation. Throughout this work, we simulate the polymer with a system of 120 sites with periodic boundary conditions. The sites’ masses equals 13 *a*.*u*.. Moreover, the initial states of the dynamical simulations are stationary solutions, provided by the self-consistent procedure previously outlined.

## Results

**Figure 1:**
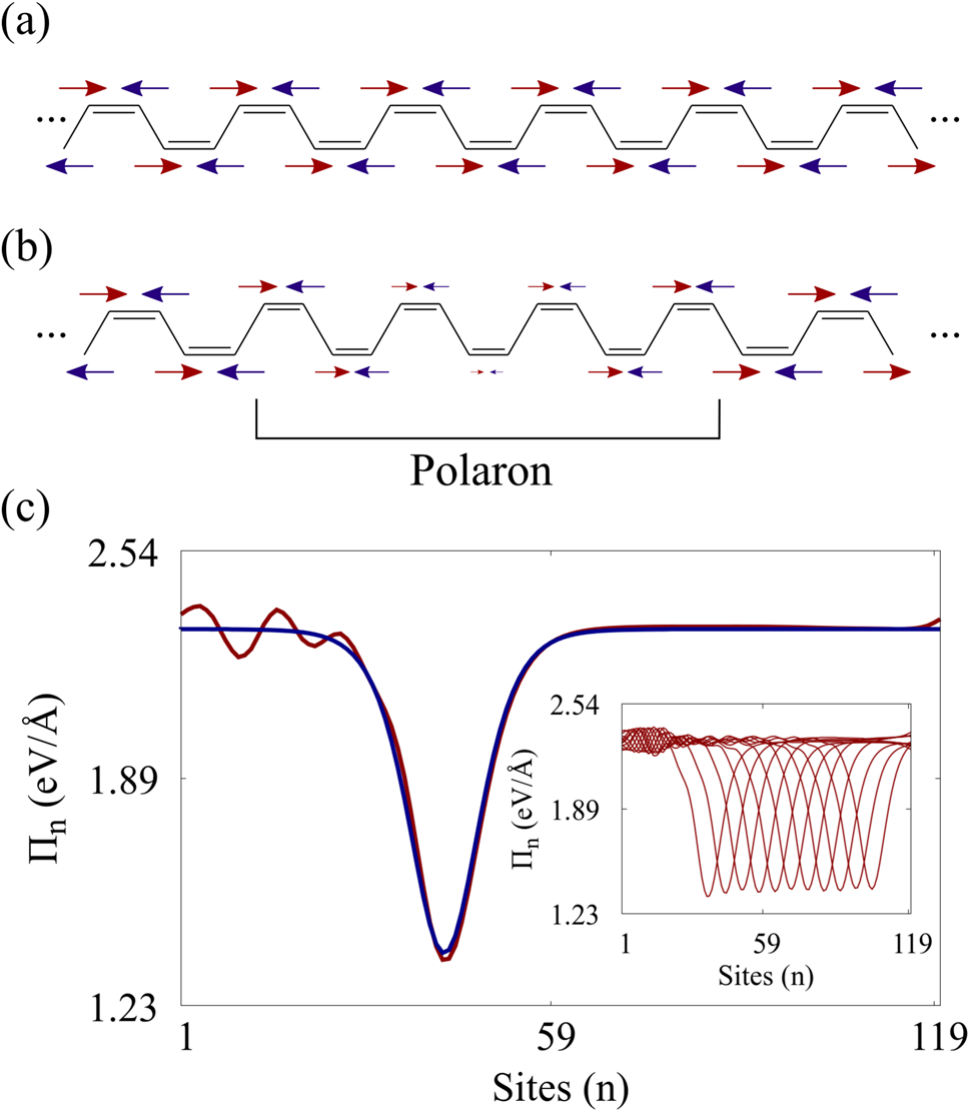
Diagrams (a) and (b) illustrate the behavior of the effective force field in two situations: (a) in the absence of quasiparticles and (b) when the chain hosts a polaron. At each site, there is a colored arrow that symbolizes the orientation of the force field caused by the $\pi$-electrons effective potential. The red color indicates odd parity, while the blue even. In (b), there is a distorted set of arrows representing the field distortion associated with the quasiparticle. In (c), the force fields, $\Pi _n$, for the conventional and alternative approaches are shown. The lines in red refer to the conventional method, while the blue one is for the effective approach. The inset presents the effective field’s time evolution as the polaron drifts.

Figure 1a represents diagrammatically the $\pi$-electron force field, $\Pi _n^d$, in the absence of any disturbance by colored arrows. One may see that sites with the same parity possess arrows of equal size, meaning they experience the same potential. Fig. 1(b) shows this field for a polaron. As can be seen, sites far away from the quasiparticle continue to feel a uniform parity-based alternating force field. However, those near it have a local distortion on the force field.

Figure 1c shows actual calculations of $\Pi _n$ of a system bearing a polaron. The conventional approach yields the red line. The effective method gives the blue one. The quasiparticle’s presence breaks the uniformity of the potential leading to a local distortion on the force field. The inset presents $\Pi _n$ at several time snapshots. The undulation on the left of the red line is due to a breather. It is dependent on how the polaron is accelerated. The breather mode is discernible around the initial position of the polaron.

The effective field is obtained by fitting $\Pi _n=\Pi _n^d+\Pi _n^p$ to a numerically calculated system with a polaron. We found that the polaron force field distortion, $\Pi _n^p$, is given by,

12 $$\begin{aligned} \Pi ^p_n=(-1)^na_1 \mathrm{{sech}^2} (a_2n-vt) \end{aligned}$$

in which, $a_1=-0.52$ and $a_2=0.27$. Here we set $v = a_{3} A(t)$ with $a_{3} = 2.7*10^{-3}$. These parameters are robust and characterize polarons in different configurations. In practice, our methodology to estimate $\Pi _{n}$ begins by explicitly simulating polaron dynamics via the usual approach. Then, with this reference result, we use Eq. 10 to find $B_{n}$, which translates into $\Pi _{n}$ through Eq. 11. Having this reference, the $\pi$ force field is determined through the fitting of the parameters in Eq. 12. The fitted expression represents the general electronic contribution to the lattice dynamics of a conjugated polymer hosting a drifting polaron driven by an external field.

**Figure 2:**
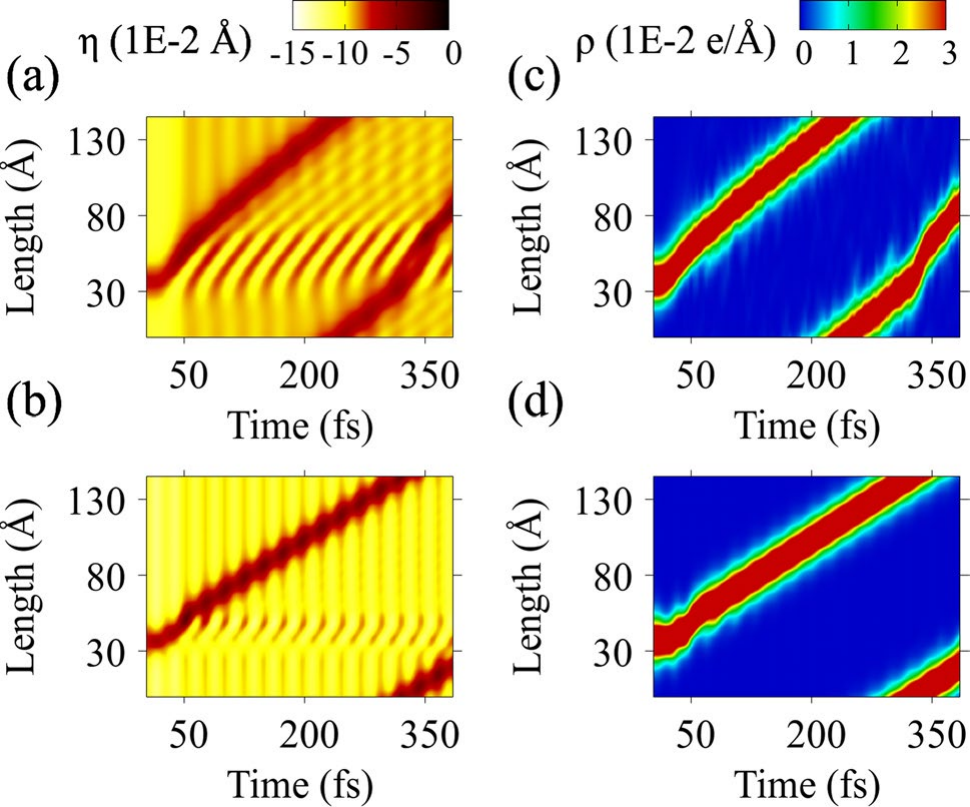
The electronic and lattice dynamics of a polaron state through the traditional and proposed model. Figures (a) and (b) refer to the lattice order parameter, $\eta _n \equiv (y_{n+1}-2y_n+y_{n-1})/4$, calculated via the conventional and the effective potential approaches, respectively. Light and dark tones indicate the distortion intensity. The localized dark-colored region refers to the polaron. On the other hand, (c) and (d) show the chain’s charge density time evolution, $\rho (t) \equiv \sum _{k,s} |\psi _{k,s}(n,t)|^2$, using the traditional procedure and the effective potential, respectively. Here, hot colors represent charge accumulation, while cold ones indicate the absence of charge.

We present in Fig. 2 the results for the system’s lattice and electronic pictures for comparison. Fig. 2a and c depict the lattice order parameter and charge density obtained through the conventional formalism. On the other hand, Fig. 2b and d refer to the same variables calculated using the effective potential.

The quasiparticle’s trajectory consists of two phases: an initial acceleration and a regime of constant velocity. The first time interval ranges from 0 to 50 fs. The profile of this period is a direct outcome of the active presence of the electric field. The polaron begins to emit phonons in both directions. After 50 fs, the electric field goes off. Right after the phonon scattering event, the quasiparticles drift with a constant velocity until about 280 fs. At this time, the distortions reach the polaron initial position due to the periodic boundary condition. As soon as the quasiparticles return to this region, the polarons of both methodologies suffer from phonon collisions since they encounter breathers excited earlier. As the two structures collide, the signature distortion of the quasiparticle is momentarily muddled^7,17^.

The trajectories delivered by both methodologies represent physically equivalent phenomena. In both cases, the polaron drifts by two distinct regimes, covering similar distances at the same time range. Additionally, the quasiparticles react identically when the acceleration ceases its influence.

Figure 2c and d show the charge density time evolution. As expected, the motion of the charged region is the same as the local deformation that appears in the lattice order parameter. As in Fig. 2a–c, at the same time, the scattered phonons collide with the polaron. These events cause a disturbance in the localized lattice distortion. Because the quasiparticle is a coupled structure of lattice distortion and charge accumulation, a similar effect occurs in (c) and (d). However, since the scattered phonons are less energetic in (d), the disturbance in the charge density profile is smaller.

There is significant accordance regarding the energy dynamics. At the start of the simulations, until about 50 fs, the electronic energy rapidly increases with the applied electric field. Next, the curve ceases to grow and begins to follow an oscillatory trajectory until the end of the simulation. In addition, one may note that polaron moves slightly faster in the traditional simulation. This occurs because, in comparison with the usual approach, the $\pi$-field formalism generates a quasiparticle with more definite lattice deformation, increasing the carrier’s transport inertia. For this reason, under same electric field conditions, the quasiparticle simulated via the usual approach will cover a slightly greater distance.

**Figure 3:**
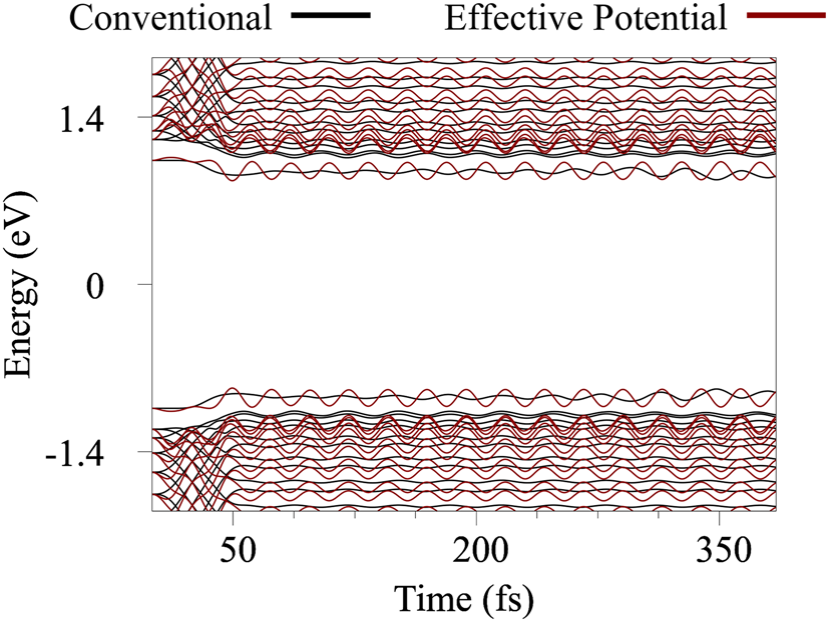
Time dependence of the electronic structure around the band gap. The red and black lines represent electronic states for $\pi$-electrons through the effective field and standard approaches, respectively. The states associated with the polaron are visible above the valence band and below the conduction band.

The equivalence between the two approaches also becomes apparent when the electronic band structure is analyzed. Figure 3 displays the time dependence of the system’s electronic band around the gap with a polaron state. Here, we present the energy states from the two independent methods. The black-colored lines refer to the energy levels obtained with the standard simulation. Alternatively, the red-colored states result from the effective potential dynamics. In both cases, the lines represent electronic states. As can be seen, the methodologies deliver equivalent electronic descriptions of the system.

The overall behavior of the bands can be summarized in two distinct regimes. The first one starts at the beginning of the simulation and goes to about 50 fs. During this period, the energy levels suffer a short but continuous shrinkage towards the center of the gap. That is a direct consequence of the Peierls instability, which is triggered by the external field. The other regime initiates right after that and goes until the end of the simulation. The energy states display small fluctuations around an average value. This profile is a result of lattice disturbances caused by the polaron’s continuous drift and the motion of scattered phonons^22^.

Besides the similarities, the band from the effective potential approach has more intense fluctuations. It is a consequence of the successive collisions with the normal oscillation mode visible in Fig. 2b. The energy bandgap of the conventional approach is found to be 1.81 ${\mp }$ 0.06 eV, while the effective field method gives 1.83 ${\mp }$ 0.1 eV.

The computation cost of the proposed model is a central point. As discussed in the methods, the approach eliminates the need to diagonalize the electronic Hamiltonian at each time step. Clearly, such optimization reduces the computational cost. All simulations were performed using the same machine. The results present a great drop in the execution time, averaging two orders of magnitude. Although such an estimate depends on the machine used, we expect no changes in the conclusion: the effective potential is two orders of magnitude faster than conventional simulations.

## Conclusion

In conclusion, we introduced an alternative model to simulate $\pi$-conjugated systems. The approach describes the electronic contribution to lattice dynamics in terms of effective $\pi$-fields. We showed that in the absence of lattice disturbances the effective force field is uniform, behaving similarly to an alternating constant gravitational field. By extending the study to excited states, we showed that the presence of quasiparticles distorts the field locally. We ran extensive tests comparing it with the usual formalism to estimate the model’s reliability. The comparison between the modelings took place with dynamical simulations of the drifting of polaron under the influence of external electric fields. The two methodologies agree on the polaron’s trajectory, the electronic band structure, and the phonon scattering. In that sense, the adoption of this new strategy causes no loss in the description’s physical accuracy. In addition, we also tested the model’s limits by enforcing different electric field regimes. Even under such conditions, the model coincides tightly with reference simulations. We also report a sharp time drop of two orders of magnitude compared with the standard approach. That is due to freeing the calculations from the constraint of diagonalization at each time in the evolution process. Our approach is an alternative to simulate much larger $\pi$-systems, where $\pi$-electron interactions are considered, and dynamics of multiple polarons become possible.

## Data Availability

The datasets used and/or analysed during the current study available from the corresponding author on reasonable request.

## References

[1] AlSalhi MS, Alam J, Dass LA, Raja M (2011). Recent advances in conjugated polymers for light emitting devices. Int. J. Mol. Sci..

[2] Bredas JL, Street GB (1985). Polarons, bipolarons, and solitons in conducting polymers. Acc. Chem. Res..

[3] Cassiano T (2020). Smooth gap tuning strategy for cove-type graphene nanoribbons. RSC Adv..

[4] da Cunha WF, Acioli PH, de Oliveira Neto PH, Gargano R, e Silva G.M. (2016). Polaron properties in armchair graphene nanoribbons. J. Phys. Chem. A.

[5] da Cunha WF, de Oliveira Neto PH, Junior LAR, e Silva G. M. (2019). Quasiparticle description of transition metal dichalcogenide nanoribbons. Phys. Rev. B.

[6] da Cunha WF, de Oliveira Neto PH, Terai A, e Silva G. M. (2016). Dynamics of charge carriers on hexagonal nanoribbons with vacancy defects. Phys. Rev. B.

[7] da Silva Pinheiro C, e Silva G. M. (2002). Use of polarons and bipolarons in logical switches based on conjugated polymers. Phys. Rev. B.

[8] de Oliveira Neto P, Teixeira J, Da Cunha W, Gargano R, e Silva G (2012). Electron-lattice coupling in armchair graphene nanoribbons. J. Phys. Chem. Lett..

[9] Duan H-G, Nalbach P, Miller RD, Thorwart M (2019). Ultrafast energy transfer in excitonically coupled molecules induced by a nonlocal peierls phonon. J. Phys. Chem. Lett..

[10] e Silva G M (2000). Electric-field effects on the competition between polarons and bipolarons in conjugated polymers. Phys. Rev. B.

[11] e Silva G M, Terai A (1993). Dynamics of solitons in polyacetylene with interchain coupling. Phys. Rev. B.

[12] Gross M, Müller DC, Nothofer H-G, Scherf U, Neher D, Bräuchle C, Meerholz K (2000). Improving the performance of doped $$\pi $$-conjugated polymers for use in organic light-emitting diodes. Nature.

[13] Guinea F (1984). Dynamics of polyacetylene chains. Phys. Rev. B.

[14] Heeger AJ, Kivelson S, Schrieffer J, Su W-P (1988). Solitons in conducting polymers. Rev. Mod. Phys..

[15] Hide JGF, Wang H (1997). Efficient photodetectors and photovoltaic cells from composites of fullerenes and conjugated polymers: Photoinduced electron transfer. Synth. Met..

[16] Jhulki S, Kim J, Hwang I-C, Haider G, Park J, Park JY, Lee Y, Hwang W, Dar AA, Dhara B (2020). Solution-processable, crystalline $$\pi $$-conjugated two-dimensional polymers with high charge carrier mobility. Chem.

[17] Johansson AA, Stafström S (2004). Nonadiabatic simulations of polaron dynamics. Phys. Rev. B.

[18] Junior LAR, Stafström S (2017). Polaron dynamics in anisotropic Holstein-Peierls systems. Phys. Chem. Chem. Phys..

[19] Kotov VN, Uchoa B, Pereira VM, Guinea F, Neto AC (2012). Electron-electron interactions in graphene: Current status and perspectives. Rev. Mod. Phys..

[20] Lee C, Wei X, Kysar JW, Hone J (2008). Measurement of the elastic properties and intrinsic strength of monolayer graphene. Science.

[21] Lee MH, Aragó J, Troisi A (2015). Charge dynamics in organic photovoltaic materials: Interplay between quantum diffusion and quantum relaxation. J. Phys. Chem. C.

[22] Lima MP, e Silva G M (2006). Dynamical evolution of polaron to bipolaron in conjugated polymers. Phys. Rev. B.

[23] Mozafari E, Stafström S (2013). Polaron dynamics in a two-dimensional Holstein-Peierls system. J. Chem. Phys..

[24] Ono Y, Terai A (1990). Motion of charged soliton in polyacetylene due to electric field. J. Phys. Soc. Jpn..

[25] Peierls R (1933). Zur theorie des diamagnetismus von leitungselektronen. Z. Phys..

[26] Pereira Junior ML, da Cunha WF, de Sousa Junior RT, Giozza WF, Silva G M, Ribeiro Junior L A (2020). Charge transport mechanism in chevron-graphene nanoribbons. J. Phys. Chem. C.

[27] Ribeiro LA, da Cunha WF, Fonseca AL, d A, e Silva G M, Stafström S (2015). Transport of polarons in graphene nanoribbons. J. Phys. Chem. Lett..

[28] Su W (1983). Soliton excitations in a commensurability 2 mixed peierls system. Solid State Commun..

[29] Su W, Schrieffer J (1980). Soliton dynamics in polyacetylene. Proc. Natl. Acad. Sci..

[30] Su W, Schrieffer J, Heeger AJ (1979). Solitons in polyacetylene. Phys. Rev. Lett..

[31] Su W-P, Schrieffer J, Heeger A (1980). Soliton excitations in polyacetylene. Phys. Rev. B.

[32] Takayama H, Lin-Liu YR, Maki K (1980). Continuum model for solitons in polyacetylene. Phys. Rev. B.

[33] Terai A, Ono Y (1991). Electric field induced depinning of a charged soliton from an impurity center in polyacetylene. J. Phys. Soc. Jpn..

[34] Xu Y, Zhang F, Feng X (2011). Patterning of conjugated polymers for organic optoelectronic devices. Small.

[35] Zhang L, Yang T, Shen L, Fang Y, Dang L, Zhou N, Guo X, Hong Z, Yang Y, Wu H (2015). Toward highly sensitive polymer photodetectors by molecular engineering. Adv. Mater..

